# Supplementary material for: Disability, pain, and wound-specific concerns self-reported by adults at risk of limb loss: A cross-sectional study using the World Health Organization Disability Assessment Schedule 2.0
Source: PLoS One. 2021 Jun 15;16(6):e0253288. doi: 10.1371/journal.pone.0253288 (PMC8205167; doi:10.1371/journal.pone.0253288)
Supplement: S1 Table — (DOCX) [file pone.0253288.s001.docx]

**S1 Table.** **Included Patient Revised Photographic Wound Assessment Tool Scores.**

| **Wound Domain – No. (%)** | **Overall**  **(n=127 with revPWAT Scored Wounds)** |
| --- | --- |
| *Size* |  |
| 0=Wound is closed (skin intact) or nearly closed (<0.3 cm^2^) | 100 (78.7) |
| 1=0.5-2.0 cm^2^ | 6 (4.7) |
| 2=2.0-10.0 cm^2^ | 5 (3.9) |
| 3=10.0-20.0 cm^2^ | 2 (1.6) |
| 4=>20.0 cm^2^ | 14 (11.0) |
| *Depth* |  |
| 0=Wound is closed (skin intact) or nearly closed (<0.3 cm^2^) | 99 (78.0) |
| 1=Full thickness | 9 (7.1) |
| 2=Unable to judge (majority of wound base covered by yellow/black eschar) | 3 (2.4) |
| 3=Full thickness involving underlying tissue layers | 12 (9.5) |
| 4=Tendon, joint capsule, bone visible/present in wound base | 4 (3.2) |
| *Necrotic tissue type* |  |
| 0=None visible or wound is closed (skin intact) or nearly closed (<0.3 cm^2^) | 105 (82.7) |
| 1=Majority of necrotic tissue is thin, white/grey, or yellow slough | 7 (5.5) |
| 2=Majority of necrotic tissue is thick, adherent white yellow slough or fibrin | 8 (6.3) |
| 3=Majority of necrotic tissue is white/grey devitalized tissue or eschar | 5 (3.9) |
| 4=Majority of necrotic tissue is hard grey to black eschar | 2 (1.6) |
| *Amount of necrotic tissue* |  |
| 0=None visible or wound is closed (skin intact) or nearly closed (<0.3 cm^2^) | 105 (82.7) |
| 1=<25% of wound bed covered | 8 (6.3) |
| 2=25-50% of wound bed covered | 7 (5.5) |
| 3=50-75% of wound bed covered | 0 (0) |
| 4=75-100% of wound bed covered | 7 (5.5) |
| *Granulation tissue type* |  |
| 0=Wound is closed (skin intact) or nearly closed (<0.3 cm^2^) | 100 (78.7) |
| 1=Majority of granulation tissue is healthy looking (even bright red appearance) | 3 (2.4) |
| 2=Majority of granulation tissue is unhealthy (pale, dull, dusky, hypergranulation) | 13 (10.2) |
| 3=Majority of granulation tissue is damaged, friable, degrading | 8 (6.3) |
| 4=There is no granulation tissue present at the base of the open wound | 3 (2.4) |
| *Amount of granulation tissue* |  |
| 0=Wound is closed (skin intact) or nearly closed (<0.3 cm^2^) | 101 (79.5) |
| 1=75-100% of open wound is covered with granulation tissue | 7 (5.5) |
| 2=50-75% of open wound is covered with granulation tissue | 7 (5.5) |
| 3=25-50% of open wound is covered with granulation tissue | 2 (1.6) |
| 4=<25% of wound bed is covered with granulation tissue | 10 (7.9) |
| *Wound edges* |  |
| 0=Wound is closed (skin intact) or nearly closed (<0.3 cm^2^) or edges are indistinct, diffuse, not clearly visible because of re-epithelialization | 101 (79.5) |
| 1=Majority of edges are attached with an advancing border of epithelium | 0 (0) |
| 2=Majority of edges are attached even with wound base (not advancing) | 12 (9.5) |
| 3=Majority of edges are unattached and/or undermined | 6 (4.7) |
| 4=Majority of edges are rolled, thickened, or fibrotic | 8 (6.3) |
| *Periulcer skin viability* |  |
| 0=None | 101 (79.5) |
| 1=One only | 2 (1.6) |
| 2=Two or three | 14 (11.0) |
| 3=Four or five | 8 (6.3) |
| 4=Six or more | 2 (1.6) |

Where majority indicates >50% and revPWAT, revised photographic wound assessment tool.
