# Supplementary material for: Disability, pain, and wound-specific concerns self-reported by adults at risk of limb loss: A cross-sectional study using the World Health Organization Disability Assessment Schedule 2.0
Source: PLoS One. 2021 Jun 15;16(6):e0253288. doi: 10.1371/journal.pone.0253288 (PMC8205167; doi:10.1371/journal.pone.0253288)
Supplement: S2 Table — (DOCX) [file pone.0253288.s002.docx]

**S2 Table.** **Correlations/Associations Between WHODAS 2.0 Scores and Wound Characteristics and Patient-Reported Pain and Disease-Specific Concern Scores.**

| **Predictor** | **r-value** | **Change in WHODAS 2.0 Score** | | **R^2^-value** |
| --- | --- | --- | --- | --- |
|  |  | **Unadjusted Estimate**  **(95% CI)** | **p-value** |  |
| Wound type | NA |  |  | 0.038 |
| Arterial |  | 1.4 (-10.8 to 13.6) | 0.82 |  |
| Venous |  | Reference | NA |  |
| Diabetic |  | 10.8 (-5.7 to 27.3) | 0.20 |  |
| Mixed |  | 2.9 (-12.0 to 17.7) | 0.70 |  |
| Postoperative |  | 8.4 (-4.3 to 21.1) | 0.19 |  |
| Other |  | 9.6 (-5.8 to 25.0) | 0.22 |  |
| Wound location | NA |  |  | 0.0073 |
| Groin |  | 0 (-26.5 to 26.5) | >0.99 |  |
| Thigh |  | Reference | NA |  |
| Leg |  | 5.3 (-9.4 to 20.0) | 0.48 |  |
| Foot or ankle |  | 2.7 (-11.2 to 16.6) | 0.70 |  |
| Other |  | -2.1 (-28.5 to 24.4) | 0.88 |  |
| Wound volume, cm^3^ | -0.11 | -0.0058 (-0.015 to 0.0034) | 0.22 | 0.011 |
| Total revPWAT score | -0.42 | -1.2 (-1.6 to -0.71) | <0.001 | 0.18 |
| Time spent concerned about their wound during the past 2-weeks | 0.26 |  |  | 0.078 |
| None of the time |  | -12.2 (-19.8 to -4.6) | 0.002 |  |
| A little of the time |  | -1.3 (-11.1 to 8.5) | 0.79 |  |
| Some of the time |  | -3.3 (-14.3 to 7.7) | 0.56 |  |
| All of the time |  | Reference | NA |  |
| Degree of discomfort or distress experienced during the past 2-weeks | 0.23 |  |  | 0.073 |
| None |  | -10.0 (-18.5 to -1.5) | 0.02 |  |
| Very little |  | -11.6 (-20.5 to -2.8) | 0.01 |  |
| A moderate amount |  | 0 (-8.7 to 8.7) | >0.99 |  |
| A great deal |  | Reference | NA |  |

Where CI indicates confidence interval; NA, not applicable; revPWAT, revised photographic wound assessment tool; and WHODAS, World Health Organization Disability Assessment Schedule.
